# A Tailored Web-based Advice Tool for Skiers and Snowboarders: Protocol for a Randomized Controlled Trial

**DOI:** 10.2196/resprot.8770

**Published:** 2018-01-17

**Authors:** Ellen Kemler, Vincent Gouttebarge

**Affiliations:** ^1^ Dutch Consumer Safety Institute (VeiligheidNL) Amsterdam Netherlands; ^2^ Academic Center for Evidence-Based Sports Medicine Academic Medical Center Amsterdam Netherlands; ^3^ Division of Exercise Science and Sports Medicine University of Cape Town Cape Town South Africa; ^4^ Amsterdam Collaboration on Health & Safety in Sports IOC Research Center Academic Medical Center/VU Medical Center Amsterdam Netherlands

**Keywords:** Winter sports, behavior, injury prevention, skiing, snowboarding

## Abstract

**Background:**

Being active in sports has many positive health effects. The direct effects of engaging in regular physical activity are particularly apparent in the prevention of several chronic diseases, including cardiovascular disease, diabetes, cancer, hypertension, obesity, depression, and osteoporosis. Besides the beneficial health effects of being active, sports participation is unfortunately also associated with a risk of injuries.

In the case of many sports injuries (eg, winter sports) preventive measures are not compulsory, which means that a behavioral change in sports participants is necessary to increase the use of effective measures, and subsequently prevent or reduce injuries in sports.

**Objective:**

The evidence-based Wintersportklaar online intervention has been developed to stimulate injury preventive behavior among skiers and snowboarders. In this article, the design of the effectiveness study will be described.

**Methods:**

A randomized controlled trial with a follow-up period of four months during the winter sport season will be conducted. The participants consist of inexperienced skiers and snowboarders. At baseline, skiers and snowboarders in the intervention and control groups are asked to report the injury preventive measures they usually take during their preparation for their winter sport holiday. One and three months after baseline, skiers and snowboarders are asked to report retrospectively in detail what measures they took regarding injury prevention during their current winter sport preparation and winter sport holiday. Descriptive analyses (mean, standard deviation, frequency, range) are conducted for the different baseline variables in both study groups. To evaluate the success of the randomization, baseline values are analyzed for differences between the intervention and control groups (chi square, independent *T* tests and/or Mann-Whitney test). Chi square tests and/or logistic regression analyses are used to analyze behavioral change according to the intention to treat principle (as initially assigned).

**Results:**

The project was funded in 2016 and enrolment was completed in 2017. Data analysis is currently under way and the first results are expected to be submitted for publication in 2018.

**Conclusions:**

To combat the negative side effects of sports participation, the use of injury preventive measures is desirable. As the use of injury prevention is usually not compulsory in skiing and snowboarding, a behavioral change is necessary to increase the use of effective injury preventive measures in winter sports.

**Trial Registration:**

Dutch Trial Registry NTR6233; http://www.trialregister.nl/trialreg/admin/rctview.asp?TC=6233 (Archived by WebCite at  http://www.webcitation.org/6wXZPzjUi)

## Introduction

Skiing and snowboarding are very popular winter sports worldwide, including among Dutch residents. Nearly one million Dutch snow sport fanatics travel each year to the mountains to spend their winter holidays in the snow, with Austria, France, Germany, Switzerland, and Italy being the most popular destinations [1]. Besides the beneficial health effects of being active, both skiing and snowboarding are also associated with a risk for musculoskeletal injuries and traumatic brain injury (42 injuries per 1,000,000 person-years for skiers and 19 injuries for snowboarders in America or 0.6 injuries per 1,000 skier days in Austria) [2-4]. Although the injury rate among skiers in Austria has decreased in recent years, attention to injury prevention should be encouraged due to the severity of these injuries [2,4].

Known risk factors for the occurrence of snow sport injuries are snow sport experience, rented equipment, weather conditions, speed, fatigue and technical errors [5,6]. The development of safer skiing environments and the use of injury preventive measures—especially a winter sport helmet—might prevent or reduce injury severity among skiers and snowboarders. However, because injury preventive measures such as helmet use for adults and wrist guard use for snowboarders in general are not compulsory on the ski slopes, a behavioral change in skiers and snowboarders is necessary to increase the use of those measures and subsequently prevent or reduce injuries in winter sports.

Accordingly, a scientific research project has started in the Netherlands (funded by ZonMW, the Netherlands Organization for Health Research and Development). The aims of this project are: (i) to develop an evidence-based intervention to stimulate injury preventive behavior among skiers and snowboarders; and (ii) to evaluate the effectiveness of the developed intervention. The effectiveness of the developed intervention will be evaluated through a randomized controlled trial. This article describes the design of this study.

## Methods

The Consolidated Standards of Reporting Trials (CONSORT) statement is followed to describe the design of the study [7]. This statement is a checklist intended to improve the quality of reports of randomized controlled trials.

### Objective and Hypothesis

The objective of the study is to evaluate the effectiveness of the Wintersportklaar intervention (www.wintersportklaar.nl; in Dutch) on injury preventive behavior among skiers and snowboarders. The hypothesis of this study is that the developed intervention would lead to a 10% increase in favorable injury preventive behavior in the intervention group in comparison to the control group.

### Study Design

**Figure 1:**
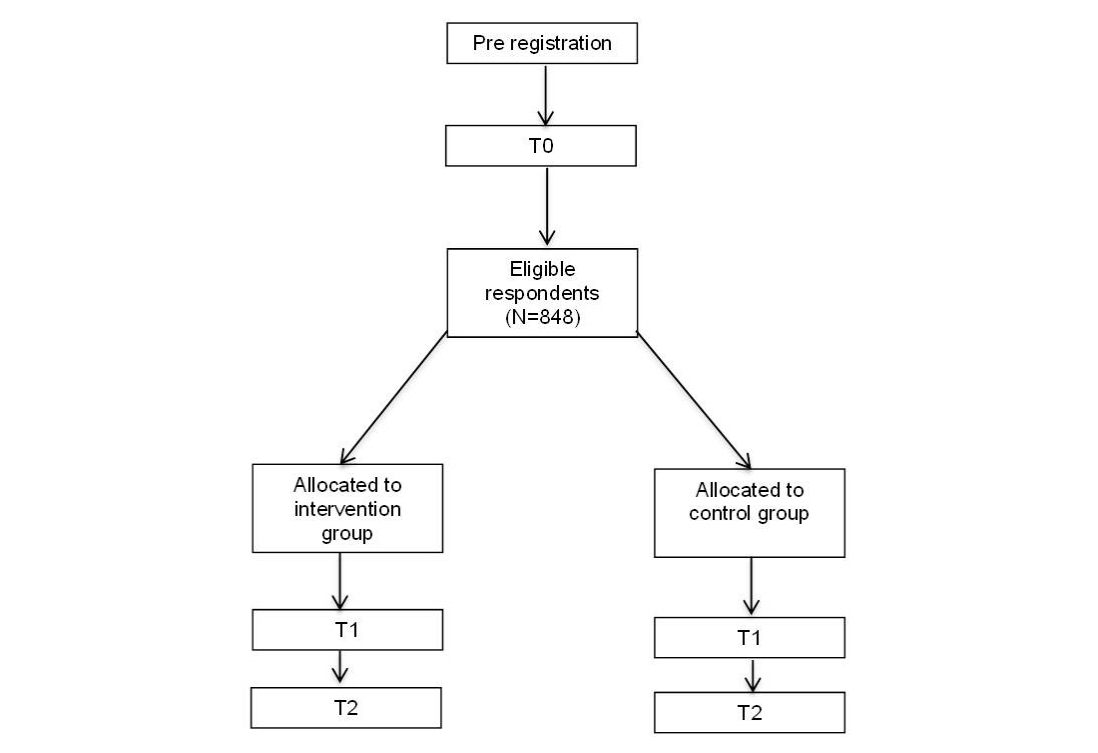
Flowchart of participants of the randomized prospective controlled trial.

A randomized controlled trial with a follow-up period of 4 months during the winter sport season will be conducted (see Figure 1). The study is registered in the Dutch Trial Registry (ID: NTR6233).

### Participants and Recruitment

The participants consist of inexperienced skiers and snowboarders. Inclusion criteria are: (i) less than five weeks of winter sport lessons or; (ii) less than two weeks of winter sport holiday per year or; (iii) less than five weeks of winter sport experience or; (iv) low physical fitness. An exclusion criterion is cross-country skiing.

Because of the relatively short duration of the winter sport season, participants can preregister 2 months before the start of the study. Participants are recruited via social media networks (ie, Facebook, websites, Twitter, LinkedIn) of the participating organizations (Dutch Consumer Safety Institute and Dutch Skiing Association). All the preregistered participants will receive the baseline questionnaire.

### Ethics, Consent and Permissions

The study protocol has been approved by the Medical Ethics Review Committee of the Academic Medical Center (W16-335 #16.417; Amsterdam, the Netherlands). Participants willing to take part in the study have to give their informed consent prior to the start of the baseline questionnaire. The flow of the participants is presented in Figure 1.

### Sample Size and Allocation

In this study, the assumption is made that a 10% increase in favorable injury preventive behavior in the intervention group compared with the control group can be achieved. To achieve 80% power with a significance level of 0.05, and taking into account a potential loss to follow-up among participants of 10% over the winter sport season, the sample size calculation reveals that 423 skiers and snowboarders per study group are needed in this study. Eligible participants will simultaneously be allocated at random to the intervention or control group after T0, using a computerized random number generator (the ASELECT function in Excel). No restrictions will be imposed for the allocation; simple randomization will be performed. All steps in the randomization process will be done by 1 researcher.

### Intervention

The Wintersportklaar intervention has been developed according to the steps of Intervention Mapping (IM) and Knowledge Transfer Scheme [8-10]. The Fogg Behaviour Model (FBM) to achieve behavior change formed the basis for this development [11]. More information on the development of Wintersportklaar is available in Kemler E, Valkenberg H & Gouttebarge V (under review).

Wintersportklaar will be made available exclusively to the participants within the intervention group, and is an evidence-based intervention developed to stimulate injury preventive behavior among skiers and snowboarders. The development of the intervention was a collaboration between snow sport experts and the target group. According to the Dutch winter sport experts who were consulted, the skill level and the physical fitness level of skiers and snowboarders are the two main dimensions involved in the risk of winter sport injuries.

Part one of the intervention is a short questionnaire. Based on 8 single questions, the physical fitness and skill level of the skiers and snowboarders will be determined and classified as: 1) low level of physical fitness and skill level; 2) low level of physical fitness and sufficient skill level; 3) sufficient level of physical fitness and low skill level; and 4) sufficient level of physical fitness and skill level. Depending on their answers, skiers and snowboarders will receive tailored advice on optimal preparation for their winter sport holiday (part two). Examples of advice include taking winter sport lessons, and strength exercises. Furthermore, advice about the use of protective gear and the 10 Fédération Internationale de Ski (FIS) rules for the conduct of winter sport safety are given. After receiving the tailored advice, the skiers and snowboarders can leave their email address on the website in order to receive a personal exercise schedule every week until the start of their winter sport holiday (part three). The Wintersportklaar intervention is made available to the participants within the intervention group. Huge efforts have been made to develop an easily accessible intervention (eg, short questionnaire, quick outcome), with a low threshold, and easily applicable injury preventive advice.

### The Control Group

The skiers and snowboarders in the control group do not have access to the intervention and prepare their winter sport holiday as they normally would.

### Injury Preventive Behavior

In our study, our main outcomes measure injury preventive behavior defined as: (i) taking winter sport lessons; (ii) performing strength exercises; (iii) the use of protective equipment; and (iv) knowledge of the 10 FIS rules for the conduct of safety in winter sport. Each injury preventive topic is divided into preparatory acts like searching for information about injury preventive measures, buying a winter sport helmet, etc, and structural injury preventive measures (eg, wearing a helmet, taking lessons). Taking winter sport lessons consists of two preparatory acts and three structural injury preventive measures. Performing strength exercises consists of two structural injury preventive measures, and the use of protective gear consists of three preparatory acts and three structural injury preventive measures. Finally, knowledge of the 10 FIS rules for the conduct of safety in winter sport consists of three preparatory acts. All injury preventive behaviors are assessed through single questions (eg, Did you buy a winter sport helmet after the start of this study? Yes/No/Not applicable).

### Procedures

At baseline, respondents will be asked about their winter sport demographic characteristics, winter sport skill level, physical fitness, current injuries and physical complaints, winter sport preparation, and winter sport injury preventive measures. Furthermore, skiers and snowboarders in the intervention and control group will be asked to report the injury preventive measures they usually take during their preparation for their winter sport holiday. One month and three months after baseline, skiers and snowboarders will be asked to report retrospectively in detail what they had done up until then regarding injury prevention during their current winter sport preparation and holiday. In the follow-up measurements, respondents will be asked about any injuries they suffered during their winter sport preparation and holidays. In our study, an injury is defined as an event during skiing or snowboarding after which the participants have to stop their winter sport activities or are unable to participate in another winter sport activity [12-15]).

For all three measurements, an online form will be sent by email by 1 researcher through SurveyMonkey, an online questionnaire system (www.surveymonkey.nl). The first reminder will be sent after 1 week if no response is received. A second reminder will be sent after 2 weeks if no response is received.

The intervention group will be additionally questioned about their use and valuation of the Wintersportklaar intervention. The evaluation of its effectiveness will not take place in an experimental setting; participants in the intervention group will be given access to the intervention, but no further conditions are applied to the use of the intervention. In the questionnaires given at one month and three months, the intervention group will be asked about their use of the intervention, the appeal of the intervention, the behavioral actions they took after being exposed to the intervention, their intention to use the intervention again, and how they would want to be informed about the intervention. If participants indicate that they do not want to use the intervention, they will be asked to explain their decision in detail.

### Statistical Analysis

Descriptive analyses (mean, standard deviation, frequency, range) are conducted for the different baseline variables for both study groups. To evaluate the success of the randomization, baseline values are analyzed for differences between the intervention and control groups (chi square, independent *T* tests and/or Mann-Whitney test).

Chi square tests and/or logistic regression analyses will be used to analyze behavioral change according to the intention to treat principle. According to this principle all participants who were enrolled and randomly allocated to the intervention or control group are included in the analysis and are analyzed in the groups to which they were randomized. In a secondary analysis, behavioral change is analyzed according to the non-intention to treat principle. The proportion of injured winter sport participants is calculated by dividing the number of injured respondents by the total number of respondents in this study and per study group. Differences in the proportion of winter sport injury between the intervention and control groups are assessed using logistic regression analysis (significance level set at *p*<.05).

## Results

The project was funded in 2016 and the funding will end in April 2018. The enrolment was completed in 2017. Currently, data analysis is under way. The first results of this randomized controlled trial are expected to be submitted for publication in 2018.

## Discussion

This article describes the design of a study that will evaluate the effectiveness of an intervention on injury preventive behavior in skiers and snowboarders. A major challenge of this study is related to the recruitment of participants and their adherence to this study. A total of 848 skiers and snowboarders will enroll for this study. To recruit respondents, several strategies are used (eg, social media such as Facebook, LinkedIn, and Twitter, and the possibility to preregister for the study). In a previous study, we also used social media to recruit skiers and snowboarders. Within two weeks, we received 300 responses, without the use of any rewards for participants or paid advertisements on social media. For this study, respondents will be offered a chance to win a money prize if they complete all three questionnaires. The chance to win a reward (€250) is used to enhance the willingness of skiers and snowboarders to participate in the study and the respondents’ adherence to the study, even after their winter sport holidays are over. In combination with the possibility to preregister for the study and the use of paid advertisements on (eg, Facebook), we believe that the recruitment of 848 skiers and snowboarders will be challenging, but feasible.

Another challenge is the use of injury preventive behavior and/or a change in injury preventive behavior as a primary outcome measure in a study regarding injury prevention rather than winter sport injuries. Clear definitions of injury preventive behavior are necessary, as well as extensive measurements of injury preventive behavior at baseline and subsequent measurements. Furthermore, we have to determine what a relevant change in injury preventive behavior is. In this study, we limit injury preventive behavior to taking winter sport lessons, performing strength exercises, the use of protective equipment, and knowledge of the 10 FIS rules of conduct. To enhance the measurement of injury preventive behavior, each injury preventive behavior topic will be operationalized in preparatory acts and structural injury preventive measures. To better detect change in our measurements, we use dichotomous answer categories instead of, for example, a 5-point scale. Finally, it is well-known that changing health-related behavior is difficult [16]. It is a lengthy process that requires small steps. Therefore, a relevant change in injury preventive behavior was set at 10%, which we think is an achievable change.

## Abbreviations

FBM: Fogg Behaviour Model
FIS: Fédération Internationale de Ski
IM: Intervention Mapping
ZonMW: Netherlands Organization for Health Research and Development
